# Ordered Mesoporous Carbon Modified with L-Arginine for Pb(II) Enrichment in Water Quality Control from Urban Roof Runoff

**DOI:** 10.3390/ma19071318

**Published:** 2026-03-26

**Authors:** Rafał Olchowski, Agnieszka Chałabis-Mazurek, Ryszard Dobrowolski

**Affiliations:** 1 Department of Pharmacology, Toxicology and Environmental Protection, Faculty of Veterinary Medicine, University of Life Sciences, Akademicka Sq. 12, 20-950 Lublin, Poland; rafal.olchowski@up.edu.pl (R.O.); agnieszka.mazurek@up.edu.pl (A.C.-M.); 2 Department of Analytical Chemistry, Faculty of Chemistry, Institute of Chemical Sciences, Maria Curie-Sklodowska University, M. C. Sklodowska Sq. 3, 20-031 Lublin, Poland

**Keywords:** CMK-3, post-synthesis modification, wastewater, lead enrichment, determination

## Abstract

Hexagonally ordered mesoporous carbon was ozonized, and the oxidized carbonaceous material was modified with L-arginine. The ozonized and L-arginine-modified carbons were extensively characterized and tested as Pb(II) ion adsorbents, with optimization of Pb(II) solution pH, exposure time, Pb(II) ion concentration and the presence of concurrent ions. Pb(II) adsorption equilibrium was achieved within 5 min at optimal pH = 2.6 or 5.3 for the oxidized and L-arginine-modified carbonaceous materials, respectively. The adsorption kinetics of both investigated materials were best described by the pseudo-first-order model. The maximum adsorption capacity for Pb(II) ions was determined to be 16 mg g^−1^ (ozonized material) or 45 mg g^−1^ (L-arginine-modified material). The Langmuir model provided the best fit for the adsorption isotherm data. Fe(III) ions mostly hindered the Pb(II) adsorption (up to 60%) on the L-arginine-modified carbon material. L-arginine-modified carbon was used to enrich Pb(II) from simulated urban roof runoff and its determination using the slurry sampling high-resolution continuum-source graphite furnace atomic absorption spectrometry technique. The developed analytical procedure was characterized by a limit of quantification of 2.63 µg L^−1^, an enrichment factor of 50, and a recovery rate of 94.8%.

## 1. Introduction

Nearly 60% of the world’s population will live in areas with insufficient freshwater resources by 2050. The problem with access to fresh water has led to the search for alternative sources. One of them is harvested rainwater (HRW), which is rainwater collected from catchment surfaces like rooftops. The quality of the HRW collected from urban rooftops depends on the roof type and age, atmospheric deposition and weather patterns [1,2,3,4]. It can contain bacteria, fungi, pesticides and various heavy metals (such as Cd, Co, Cr, Ni and Pb). Typically, the HRW pH ranges from 4.7 to 7.1, depending on its composition. An example composition of HRW is as follows: Cl^−^ (2.8 mg L^−1^), SO_4_^2−^ (2.8 mg L^−1^), NO_3_^−^ (0.8 mg L^−1^), PO_4_^3−^ (0.2 mg L^−1^), SiO_2_ (0.8 mg L^−1^), Ca (1.7 mg L^−1^), Cu (18.9 µg L^−1^), K (0.8 mg L^−1^), Mg (290 µg L^−1^), Na (0.8 mg L^−1^), Ag (76.1 µg L^−1^), Al (41.2 µg L^−1^), Ba (16.8 µg L^−1^), Mo (30.7 µg L^−1^), Sb (3.1 µg L^−1^), Zn (2.6 mg L^−1^), and Pb (7.4 µg L^−1^) [3]. Over 80% of the heavy metals present in a combined sewage system may originate from urban roof runoff. The chronic consumption of HRW contaminated with heavy metals, with concentrations that can exceed the values stated for drinking water quality standards (like 10 µg L^−1^ for Pb and 6 µg L^−1^ for Mo according to the World Health Organization guidelines) [3], can cause serious health problems, like tumor development and cardiovascular and metabolic diseases. Thus, monitoring heavy metal concentrations in HRW could become indispensable.

Various analytical techniques can be applied for the determination of Pb traces in HRW: inductively coupled plasma mass spectrometry (ICP-MS), inductively coupled plasma optical emission spectrometry (ICP-OES), graphite furnace atomic absorption spectrometry (GF AAS), etc. ICP-MS provides the best sensitivity and selectivity for Pb analysis in environmental samples, but its high cost is inseparable from the technique. Thus, other low-cost analytical techniques can be used for this purpose, like GF AAS. This technique is commonly used in analytical laboratory practice. In order to improve the analytical parameters of the procedure using GF AAS detection, a preconcentration/enrichment step is introduced into the overall analytical procedure. This additional step in the analytical procedure can lead to interference removal and signal enhancement. The enrichment of Pb from the environmental sample can be performed using the low-cost solid-phase extraction (SPE) technique. Sorbents used in SPE are characterized by a high specific surface area and tunable surface functionalities, which provide selectivity towards the analyte and high enrichment efficiency. Another crucial parameter of SPE sorbents is their degree of purity, which helps prevent increases in background noise [5,6,7]. In SPE, different porous solids can be applied: ordered mesoporous silicas [8], ion-imprinted polymers [9], activated carbons [10,11], carbon nanotubes [5,12], and graphene oxides [13,14]. Ordered mesoporous carbons (OMCs) can also be used for this purpose.

One of the OMCs’ family representatives is the CMK-3 mesoporous carbon (Carbon Mesostructured by KAIST No. 3). CMK-3 is prepared via a hard-templating route using the SBA-15 (Santa Barbara Amorphous No. 15) silica template and a carbon source (e.g., sucrose). This carbonaceous material is characterized by hexagonally ordered mesoporous channels and a highly porous hydrophobic surface (specific surface area > 900 m^2^ g^−1^). The ordered mesochannels of this material enable fast analyte transport from the sample solution to the carbon-based surface, thereby considerably shortening the enrichment step. The highly porous surface of the CMK-3-type carbonaceous material can be readily chemically modified with inorganic and organic functionalities, thereby providing a large number of surface active sites. It can give the material selectivity towards the heavy-metal analyte and improve its adsorption capacity, influencing the enrichment factor [15,16,17].

In this work, for the first time, the pristine CMK-3-type carbonaceous material was chemically modified by L-arginine after its ozonation. The prepared materials were characterized using various physicochemical techniques. The adsorption of Pb(II) ions from aqueous solutions onto the investigated materials was optimized by examining the effects of pH, contact time, Pb(II) concentration, and the presence of competing ions. The experimental adsorption kinetics and equilibrium data were analyzed using pseudo-first-order, pseudo-second-order, and Elovich models for kinetics, and Langmuir, Freundlich, and Langmuir–Freundlich models for adsorption isotherms. Also, regeneration studies of the Pb-loaded carbonaceous material were conducted using the following liquid media: HCl_(aq)_, HNO_3(aq)_, and NaHCO_3(aq)_. Finally, the L-arginine-modified CMK-3-type carbonaceous material proved effective for the enrichment of the Pb(II) traces from HRM samples before their determination by the slurry sampling high-resolution continuum source GF AAS technique.

## 2. Materials and Methods

### 2.1. Chemicals and Materials

The pristine hexagonally ordered mesoporous carbon of the CMK-3 type (denoted as PMC) was used as the basic adsorbent for subsequent physicochemical modifications. Its synthesis conditions were described in our previous work [18].

The ozone used for carbon oxidation was produced by an ozone generator (ZY-H102, Garbów, Poland) with an efficiency of 500 mg O_3_ hr.^−1^. Other reagents used during our study were as follows: HNO_3_ (65%, Suprapur, Merck, Darmstadt, Germany), NaOH (>95%, Fluka, Buchs. Switzerland), Fe(NO_3_)_3_ · 9H_2_O (p. a., Merck, Darmstadt, Germany), NaHCO_3_ (>99%, Pol-Aura, Poland), KCl (>99%, Merck, Darmstadt, Germany), NaNO_3_ (>99%, Merck, Darmstadt, Germany), Na_2_SO_4_ (>99%, Merck, Darmstadt, Germany), Mg(NO_3_)_2_ (>99%, Merck, Darmstadt, Germany), Ca(NO_3_)_2_ · 4H_2_O (>99%, Merck, Darmstadt, Germany), Na_3_PO_4_ (>99%, Merck, Darmstadt, Germany), L-arginine (>99%, Pol-Aura, Poland) and Pb standard solution (Pb(NO_3_)_2_, 1 g L^−1^, Merck, Darmstadt, Germany). All experiments were carried out using double-distilled Milli-Q water from Millipore (Merck, Darmstadt, Germany).

### 2.2. Carbon Modification Procedures

#### 2.2.1. Ozonation

**Figure 1:**
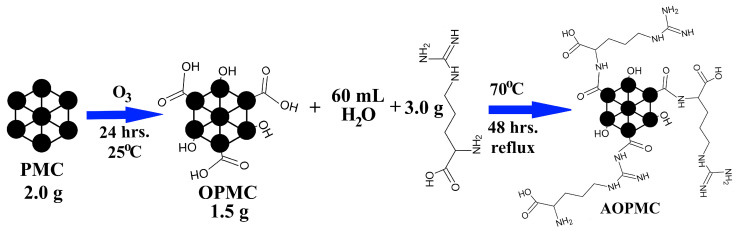
The modification scheme of the PMC carbonaceous material.

The pristine CMK-3-type carbon ozonation procedure was described in detail in our previous work [17]. The obtained material was denoted as OPMC (Figure 1).

#### 2.2.2. L-Arginine Modification

A total of 1.5 g of the OPMC material was put in the round-bottom flask. Next, the aqueous solution of L-arginine (3 g L-arginine in 60 mL of deionized water) was mixed with the material. The obtained carbonaceous slurry was heated in reflux at 70 °C for 48 h. After that time, the slurry was filtered, and the carbonaceous material was rinsed with distilled water until the filtrate became neutral (pH ≈ 7). The material was dried in the laboratory oven (SML 32/250, Zalmer, Rzeszów, Poland) at 120 °C for 24 h. The L-arginine-modified carbon was denoted as AOPMC (Figure 1).

### 2.3. Instrumentation

The porosity measurements of the studied carbonaceous materials were performed using an ASAP 2420 analyzer (Micromeritics Inc., Norcross, GA, USA) at −196 °C. Before the analysis, the samples were degassed under vacuum at 120 °C for 12 h. The textural parameters, including BET surface area (S_BET_ [m^2^ g^−1^]), total pore volume (V_T_ [cm^3^ g^−1^]), and BJH pore diameter (d_BJH_ [nm]), were determined from the desorption branch of the nitrogen adsorption–desorption isotherm.

The morphology of the carbonaceous particles and their elemental composition to a depth of several µm were examined using a scanning electron microscope (SEM) (Carl Zeiss Ultra Plus, Carl Zeiss, Jena, Germany) equipped with a BrukerAXS energy-dispersive X-ray (EDX) detector (Bruker, Karlsruhe, Germany). The microscope was additionally fitted with secondary electron (SE) and backscattered electron (BSE) detectors. The analyses were carried out at an acceleration voltage of 20 kV and a probe current of 5 nA.

The hexagonal ordering of mesopores in the studied carbons was evaluated using X-ray diffraction (XRD) patterns obtained with an Empyrean diffractometer (PANalytical, Malvern, UK) equipped with a CuKα radiation source. The measurements were performed with a step size of 0.02° and a counting time of 10 s per step.

The defect density in the graphene sheets of the studied carbons was analyzed by Raman spectroscopy using a dispersive Raman microscope (inVia Reflex, Renishaw, Wotton-under-Edge, UK) equipped with a 514 nm ion–argon laser (20 mW).

The zeta potential and acid–base properties of the studied samples were determined using a Zetasizer Nano ZS (Malvern Instruments, Malvern, UK) and a CP-401 pH meter (Elmetron, Zabrze, Poland) equipped with a glass electrode. For the zeta potential measurements, 2 mg of the carbon sample was dispersed in 2 mL of a 0.001 mol L^−1^ KCl solution. The acid–base properties of the carbonaceous materials were evaluated by measuring the pH of 5 mL of 0.001 mol L^−1^ KCl solution after contact with 20 mg of the carbon material for 24 h.

The CHN elemental composition was determined using an EA 3000 Elemental Analyzer (Euro Vector, Milan, Italy).

Surface functional groups of the studied carbons were identified using Fourier-transform infrared (FT-IR) and X-ray photoelectron spectroscopy (XPS). FT-IR spectra were recorded with a Nicolet 8700A spectrometer (Thermo Scientific, Waltham, MA, USA) in the 400–4000 cm^−1^ range using KBr pellets. XPS analysis was performed using a Multi-Chamber Analytical System (Prevac, Rogów, Poland) equipped with monochromatic Al Kα radiation (1486.6 eV) (Gammadata Scienta, Uppsala, Sweden) with an X-ray power of 450 W. The C1s peak at 285 eV was used as the reference for binding energy calibration.

Pb concentration in the aqueous phase was determined using a SpectrAA 880 flame atomic absorption spectrometer (FAAS) (Varian, Melbourne, Australia) equipped with a Pb hollow cathode lamp. The operating conditions were as follows: lamp current 10 mA, wavelength 217.0 nm, slit width 1.0 nm, air flow 13.5 L min^−1^, acetylene flow 2.0 L min^−1^, and burner height 13.5 mm.

The Pb determination in carbonaceous slurries was performed by the slurry sampling high-resolution continuum source graphite furnace atomic absorption spectrometer (SS HR-CS GF AAS) (ContrAA 700, Analytik Jena AG, Jena, Germany) equipped with the high-pressure xenon short-arc lamp working in a hot spot mode (GLE, Berlin, Germany). The measurement conditions were as follows: wavelength: 217.001 nm, pyrolysis temperature: 1000 °C, atomization temperature: 1500 °C, chemical modifier: 1 wt. % NH_4_H_2_PO_4_, atomization time: 5s, integration time: 5s, integration: CP (central pixel) ± 1, sampling volume: 20 µL, modifier volume: 5 µL, mathematical background correction using the background correction pixels. The calibration was performed using aqueous Pb standard solutions with Pb concentrations in the range 4–40 µg L^−1^.

### 2.4. Static Pb(II) Adsorption Studies

A total of 20 mg of OPMC or AOPMC was contacted with 5 mL of an aqueous Pb(II) solution of known concentration and adjusted pH. The resulting carbon suspensions were agitated at 170 rpm for 24 h, except during the adsorption kinetics experiments. After reaching equilibrium, the suspensions were centrifuged to separate the aqueous phase from the solid material. The Pb(II) concentration in the solution before and after adsorption was determined by FAAS. The Pb(II) adsorption (A_Pb(II)_ [mg/g]) was calculated as follows:

(1) $$A_{Pb(II)}=\frac{\left(C_{in.}-C_{eq}\right)V}{m}$$

where C_in_. [mg L^−1^] is the starting Pb(II) concentration, C_eq_ [mg L^−1^] is the equilibrium Pb(II) concentration, V [mL] is the volume of the adsorbate solution, and m [mg] is the mass of the adsorbent.

Pb(II) adsorption onto the studied carbons was optimized by evaluating the effects of solution pH (1–5), contact time (5–1440 min), and Pb(II) concentration (4–500 mg L^−1^). The pH of the Pb(II) solution (48 mg L^−1^) was adjusted using 1 mol L^−1^ HNO_3_ or 1 mol L^−1^ NaOH. The effect of contact time on Pb(II) adsorption was investigated at an initial pH of 5.0 and a Pb(II) concentration of 49 mg L^−1^. Adsorption isotherms were determined at pH 5.0, a temperature of (25 ± 4) °C, and a contact time of 24 h.

Pb(II) adsorption kinetics were analyzed using three theoretical models: pseudo-first-order (PFO) (Equation (2)), pseudo-second-order (PSO) (Equation (3)), and the Elovich model (Equation (4)) [17,19]. The corresponding equations are given below:

(2) $$g_{t}=g_{eq}\left(1-e^{-k_{1}t}\right)$$

(3) $$g_{t}=\frac{g_{eq}^{2}k_{2}t}{1+g_{eq}k_{2}t}$$

(4) $$g_{t}=\frac{1}{\beta }\ln\left(\alpha \beta \right)+\frac{1}{\beta }\ln t$$

where g_t_ [mg g^−1^] is the amount of Pb(II) adsorbed at time t, g_eq_ [mg g^−1^] is the equilibrium adsorption capacity, and t [min] denotes the adsorption time. The constants k_1_ [min^−1^] and k_2_ [g mg^−1^ min^−1^] represent the rate constants of the pseudo-first-order and pseudo-second-order models, respectively. The parameter α [mg g^−1^ min^−1^] corresponds to the initial adsorption rate, while β [g mg^−1^] is associated with the chemisorption activation energy and surface coverage.

Pb(II) adsorption isotherms were analyzed using three theoretical models: Langmuir (Equation (5)), Freundlich (Equation (6)), and Langmuir–Freundlich (Equation (7)) [17,20]. The corresponding mathematical expressions are given below:

(5) $$g_{eq}=\frac{C_{eq}g_{m}k_{L}}{1+C_{eq}k_{L}}.$$

(6) $$g_{eq}=k_{F}C_{eq}^{n_{F}}$$

(7) $$g_{eq}=\frac{g_{m}{\left(C_{eq}k_{LF}\right)}^{n_{LF}}}{1+{\left(C_{eq}k_{LF}\right)}^{n_{LF}}}$$

where g_m_ [mg g^−1^] is the maximum Pb(II) adsorption capacity, C_eq_ [mg L^−1^] is the equilibrium Pb(II) concentration, and k_L_ [L mg^−1^] denotes the Langmuir equilibrium constant. The constants k_F_ [mg^(1−nF)^ L^nF^] and k_LF_ [L mg^−1^] represent the Freundlich and Langmuir–Freundlich equilibrium constants, respectively, while n_F_ and n_LF_ are the corresponding dimensionless model parameters.

The effect of competing ions on Pb(II) adsorption onto the AOPMC material was investigated in the presence of HCO_3_^−^, Cl^−^, SO_4_^2−^, PO_4_^3−^, NO_3_^−^, Mg^2+^, Ca^2+^, and Fe^3+^ ions. For this purpose, 20 mg of AOPMC carbon was mixed with 5 mL of an aqueous solution containing Pb(II) ions (40 mg L^−1^) and competing ions within the concentration ranges of 0–1000 mmol L^−1^ for anions and 0–200 mg L^−1^ for cations. The pH of the solutions was adjusted to 3.0. After shaking the suspensions for 24 h, the initial and equilibrium Pb(II) concentrations were determined, and the adsorption capacity was calculated as described above. Finally, the relative adsorption was calculated according to Equation (8):

(8) $$A_{100\%}=\frac{A_{Pb(II)}}{A_{max.}}100\%$$

where A_100%_ [%] is the relative adsorption of Pb(II) onto AOPMC carbon, whereas A_max_ [mg g^−1^] denotes the maximum Pb(II) adsorption achieved in the investigated system.

### 2.5. Regeneration

Pb desorption from Pb-loaded AOPMC carbon was investigated using three eluents (HCl, HNO_3_, and NaHCO_3_) at concentrations of 0.01–1.00 mol L^−1^. Briefly, 5 mg of Pb-loaded AOPMC (41.2 mg g^−1^) was mixed with 2 mL of the desorbing solution and shaken for 24 h. The suspension was then centrifuged and the Pb concentration in the supernatant was determined by FAAS. The desorption degree of Pb from AOPMC was calculated using Equation (9):

(9) $$O_{Pb}=\frac{C_{Pb}V_{lm}}{m_{des.}A_{Pb_{carb}.}}100\%$$

where O_Pb_ [%] represents the degree of Pb desorption from the investigated material, C_Pb_ [mg L^−1^] is the Pb concentration in the liquid phase after the desorption process, and V_lm_ [mL] denotes the volume of the liquid medium used. The parameter m_des_ [mg] corresponds to the mass of the Pb-loaded adsorbent, while A_Pb_ [mg g^−1^] indicates the Pb content in the Pb-loaded AOPMC carbon.

### 2.6. Application

The AOPMC carbonaceous material was applied for the Pb(II) enrichment from the simulated urban runoff samples prior to determination by the SS-HR-CS GF AAS technique. Three simulated urban runoff samples were obtained by appropriate dilution of a Pb standard solution to concentrations of 4, 8, and 12 µg L^−1^ in the matrix reference material (Rain-97, National Water Research Institute of Canada) [21] (Table S1). AOPMC carbon (20 mg) was added to the 50 mL of the simulated urban runoff sample with adjusted pH_eq_ = 5.3. The carbonaceous slurry was shaken for 24 h. Then the Pb-loaded AOPMC carbon was centrifuged, filtered, and dried at 120 °C for 24 h. Next, 20 mg of Pb-loaded AOPMC was added to 1 mL of HNO_3_ (0.1 mol L^−1^), and the resulting slurry was homogenized in a Vortex directly before measurements with SS HR-CS GF AAS. The Pb concentration in the analyzed urban runoff wastewater was calculated according to the following equation (Equation (10)):

(10) $$Z_{Pb}=\frac{C_{Pb_{det}.}V_{med.}m_{1}}{m_{2}V_{sample}}$$

where Z_Pb_ [µg L^−1^] is the Pb concentration in the analyzed sample, C_Pbdet_. [µg L^−1^] is the Pb concentration determined in the carbonaceous slurry, V_med_. [mL] is the volume of the 0.1 mol L^−1^ HNO_3_ used for the carbonaceous slurry preparation, V_sample_ [mL] is the analyzed sample volume, m_1_ [mg] is the AOPMC mass used for Pb adsorption from the analyzed sample and m_2_ [mg] is the Pb-loaded AOPMC mass used for the slurry preparation. Three replicates were made for each sample. The recovery was estimated using the 1640 reference material (Trace Elements in Natural Water, NIST) [22] by the proposed analytical procedure.

## 3. Results and Discussion

### 3.1. Physico-Chemical Properties

**Figure 2:**
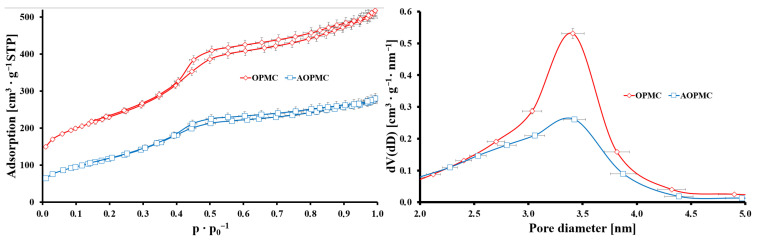
N_2_ adsorption/desorption isotherms and PSDs for synthesized carbons (error bars—precision from triplicate measurements).

The porosity of the studied carbons was determined based on low-temperature N_2_ adsorption/desorption isotherms and pore size distribution (PSD) (Figure 2). The isotherms of both materials could be classified as the IVa type with H1 type hysteresis loop starting at *p* p_0_^−1^ = 0.4, which was typical for narrow-mesopored (3.4 nm) carbons of CMK-3 type (Table 1) [17]. Additionally, both carbon materials possessed a large specific surface area (>400 m^2^ g^−1^), consistent with their developed microporous structure. Twice as high nitrogen adsorption values for OPMC as for AOPMC corresponded to the substantial change in the specific surface area after L-arginine modification (813 m^2^ g^−1^ and 430 m^2^ g^−1^ for OPMC and AOPMC, respectively). This change was also observed for total pore volumes of studied samples (0.82 cm^3^ g^−1^ (OPMC) and 0.43 cm^3^ g^−1^ (AOPMC)) (Table 1). Noticeable changes were also observed in the PSDs of the studied carbons. Probably, L-arginine molecules and their polymeric derivatives could fill part of the micro- and mesoporous structure of the modified carbonaceous material.

In the Raman spectra of the studied materials, two bands were observed: the D band (defects in graphene domains) at 1317 cm^−1^ and the G band (graphene domains without defects) at 1581 cm^−1^ [17]. The intensity ratio of these two bands (I_D_/I_G_) for both carbonaceous materials was similar and lower than 1.0 (0.86 for OPMC and 0.87 for AOPMC) (Table 1). It could be the result of the incorporation of L-arginine molecules and their polymers, rather than on the edges of graphene domains, than inside its structure.

The L-arginine modification of OPMC carbon resulted in the shift of pH_PZC_ value towards pH = 7 (change from 2.90 to 6.53). Simultaneously, the zeta potential values have been practically preserved (−33.7 mV for OPMC and −33.1 mV for AOPMC) (Table 1). The increase in pH_PZC_ may be attributed to the introduction of basic amine functionalities on the carbon surface. Some of them could react with the carboxylic groups, creating CONH- bonds. Moreover, L-arginine molecules possessed both amine and carboxylic groups, so some carboxylic groups could still be present on the AOPMC surface. Thus, the carbonaceous surface was negatively charged even after amino acid modification.

**Figure 3:**
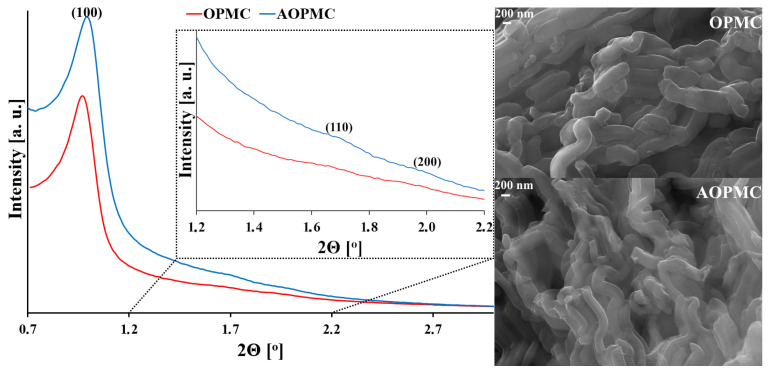
XRD patterns and SEM microphotographs (magn. 50 kx) for the studied carbons.

In Figure 3, XRD patterns and SEM microphotographs of the studied carbonaceous materials are presented. For both materials, three reflections were observed at 2θ angles of 1.0°, 1.7°, and 1.9°, which were associated with the hexagonal order of mesopores (p6 mm), similar to CMK-3-type carbon [23]. Modification of OPMC carbon with L-arginine did not affect the degree of mesopore ordering. Moreover, both carbon materials exhibited a rod-like morphology [24]; therefore, the applied surface modification did not affect their microstructure.

**Figure 4:**
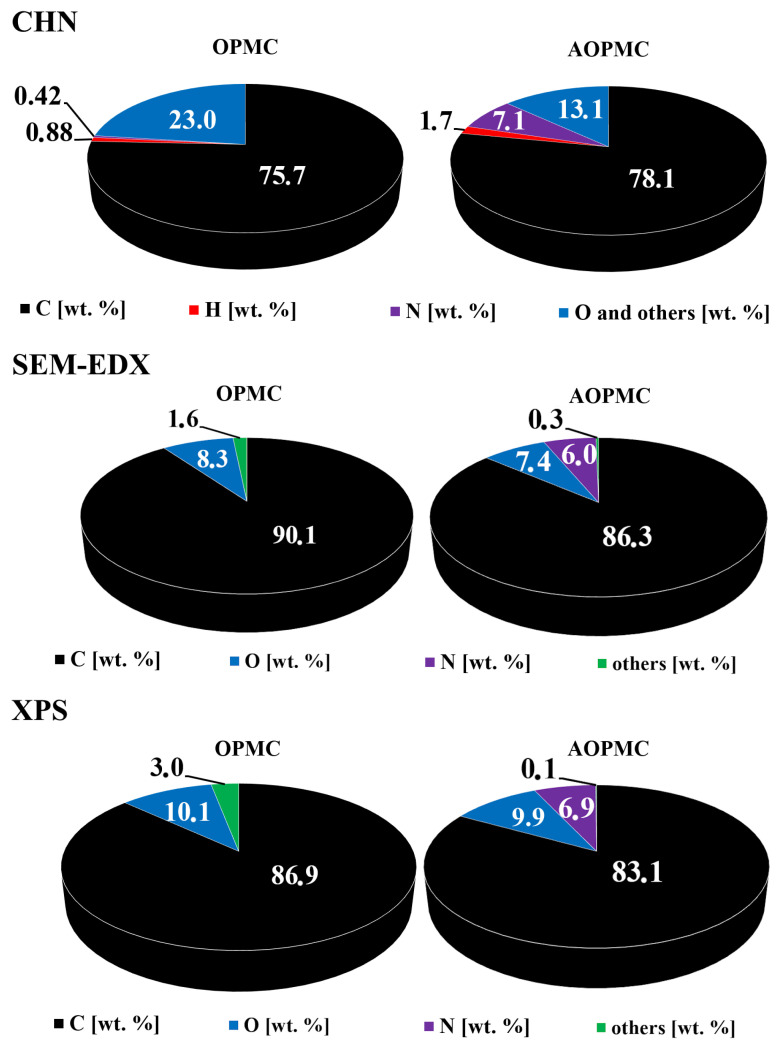
Elemental composition of the studied samples.

The elemental composition of OPMC and AOPMC materials studied by CHN, SEM-EDX and XPS techniques is presented in Figure 4. The carbon was the main constituent of both materials (75.7–90.1 wt. % (OPMC) and 78.1–86.3 wt. % (AOPMC)). These materials also contained heteroatoms such as oxygen (8.3–10.1 wt. % (OPMC) and 7.4–9.9 wt. % (AOPMC)) and nitrogen (0.42 wt. % (OPMC) and 6.0–7.1 wt. % (AOPMC)), as well as other elements. The L-arginine modification of the OPMC material successfully provided the introduction of nitrogen onto the carbonaceous surface with practically no substantial change in the oxygen content, because of the presence of both elements in L-arginine molecules. Higher values obtained by XPS suggested more oxygen and nitrogen groups on the carbonaceous surface than in the deeper layers of the material. It can be assumed that the L-arginine modification of the OPMC material was based on the L-arginine molecules’ adsorption on the OPMC surface and/or chemical binding of the modifier molecules to the carbonaceous surface by the formation of -CONH- bonds.

**Figure 5:**
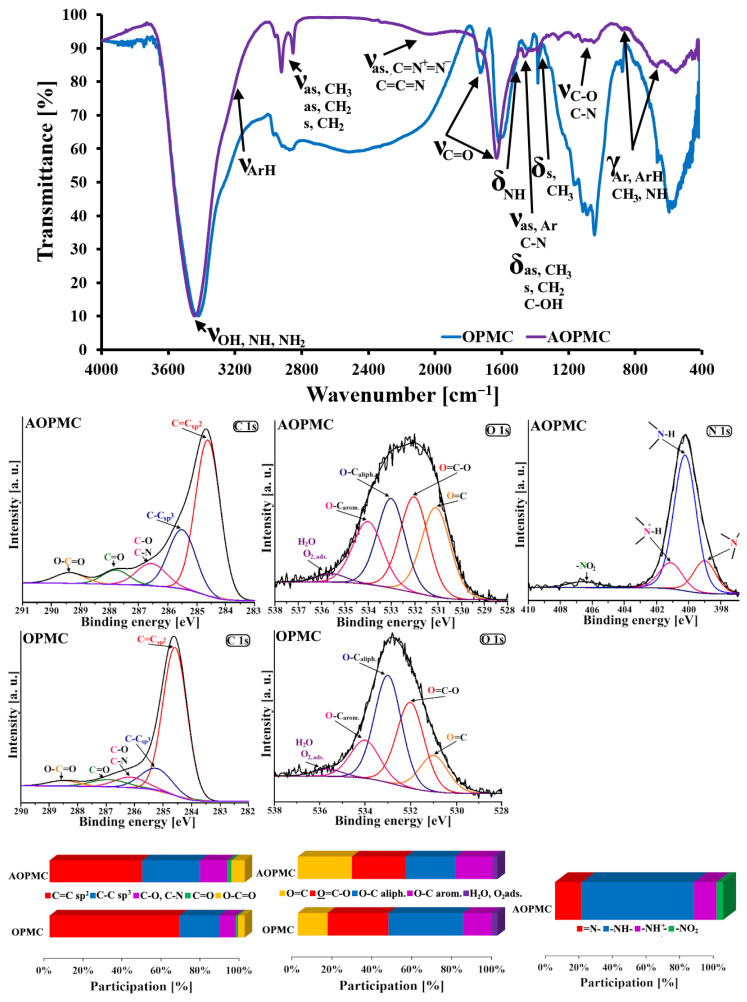
Surface analysis of the studied samples using FT-IR and XPS.

In Figure 5, the results of surface studies on both carbonaceous samples, performed using FT-IR and XPS, are presented. On both FT-IR spectra the following bands are presented: ν_OH, NH, NH2_ (3443 cm^−1^), ν_ArH_ (3200–3000 cm^−1^), ν_as, CH3_ (2963 cm^−1^), ν_as, CH2_ (2919 cm^−1^), ν_s, CH2_ (2851 cm^−1^), ν_C=O_ (1746 cm^−1^, 1629 cm^−1^), ν_as, Ar; C-N_ and δ_as, CH3; s, CH2; C-OH_ (1470–1427 cm^−1^), δ_s, CH3_ (1381 cm^−1^), ν_C-O, C-N_ (1313–1050 cm^−1^) and γ_Ar, ArH, CH3, NH_ (882–678 cm^−1^) [25]. Additionally, during the L-arginine modification of the OPMC carbonaceous material the band ν_as, C=C=O_ (2124 cm^−1^) vanished and the bands ν_C=C=N; as, C=(N+)=(N-)_ (2045 cm^−1^) and δ_NH_ (1507 cm^−1^) appeared [17,25]. The appearance of the FT-IR bands corresponding to N-containing groups confirms the incorporation of this element into the AOPMC surface. Moreover, the significant intensity decrease of bands related to the OH, C-O, C=O (at higher wavelengths), CH_2_, CH_3_, ArH, Ar and the simultaneous vanishment of the C=C=O band is probably caused by the creation of -CONH- between the OPMC surface and the L-arginine species during the chemical modification of the carbonaceous surface. Additionally, the large-sized surface functionalities (i.e., rings arginine-carbonaceous surface) could be formed during the modification process of the OPMC carbonaceous material due to the chemical interactions between L-arginine species and the surface oxygen-containing functionalities of the OPMC carbon. These large-sized surface functionalities could be responsible for the vibration damping of the CH_3_, CH_2_, Ar and ArH groups.

The detailed XPS spectra of the C1s, O1s, and N1s regions were also recorded (Figure 5). Deconvolution of the C1s signal for both materials revealed five components: C=C sp^2^ (284.6 eV), C-C sp^3^ (285.5 eV), C-O/C-N (286.5 eV), C=O (287.7 eV) and O-C=O (284.6 eV) [26]. In the case of the O1s signal, five peaks were identified at the following binding energies: 530.9 eV (O=C), 531.9 eV (O=C-O), 533.0 eV (O-C_aliph_.), 534.0 eV (O-C_arom_.) and 535.6 eV (H_2_O, O_2ads_.) [27,28]. Additionally, for AOPMC carbonaceous material the deconvolution of the N1s signal provided 4 peaks located at the following binding energies: 398.9 eV (=N-), 400.0 eV (-NH-), 400.9 eV (-NH^+^-) and 406.3 eV (-NO_2_) [29]. According to the deconvoluted peaks’ participation in the appropriate XPS core energy signal intensities for the studied materials, the following statements were formulated. During the chemical modification of the OPMC surface by the L-arginine, the participation of the C=C sp^2^ peak in the C1s intensity decreased (from 66.7% to 47.5%). Simultaneously, the participation increase in the C1s intensity was observed for C-C sp^3^ (from 20.4% to 29.6%), C-O/C-N (from 8.4% to 14.1%), C=O (from 0.8% to 2.0%) and O=C-O (from 3.8% to 6.8%). In the case of O1s signal, the participation of its intensity increased for O=C (from 15.0% to 27.2%) and O-C_arom_. (from 14.4% to 18.7%), and it decreased for O=C-O (from 30.5% to 27.0%) and O-C_aliph_. (from 37.4% to 24.7%). The content increase of the C atoms located in aliphatic chains and C-N groups could be the result of the incorporation of the arginine in the AOPMC structure. In turn, the content increase of the O atoms located in C=O and the content increase of the C atoms presented in C-N groups, along with the simultaneous content decrease of the O atoms located in O-C_aliph_. Groups, could be caused by the formation of -CONH- groups as a result of the reaction between the surface -COOH/-OH groups of OPMC material and amine groups of the arginine. Additionally, the content decrease of the O atoms presented in O=C-O and the simultaneous content increase of these atoms located in O-C_arom_. groups could be due to the reaction between the aromatic carbon rings and arginine, leading to the formation of =N- groups (16.0% contribution to the N1s signal intensity). Moreover, the highest participation of N1s signal intensity for AOPMC material was observed for -NH- groups (66.7%) present in arginine, its condensates and/or -CONH- bonds.

### 3.2. Pb(II) Adsorption Optimization

**Figure 6:**
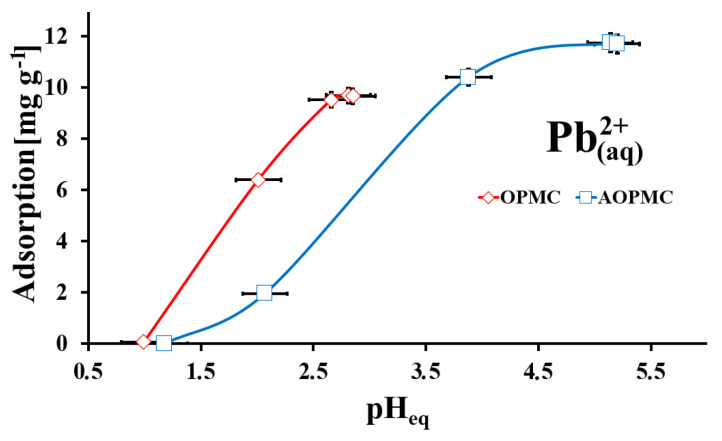
The aqueous solution pH influence on Pb(II) adsorption onto studied materials (m/V = 4 g L^−1^, C_in_Pb(II)_ = 48 mg L^−1^, t = 24 h; error bars—precision from triplicate measurements).

The effect of the pH of the Pb(II) solution on the adsorption of Pb(II) ions onto the surface of OPMC and AOPMC carbonaceous materials was studied (Figure 6). For both studied materials, the tendency was similar: the higher Pb(II) adsorption in the studied adsorption systems due to the pH rise of the solution up to the exact pH_eq_ value (optimal), in which the maximum uptake of Pb(II) was obtained (10 mg g^−1^ for OPMC at pH_eq_ = 2.6 and 11.8 mg g^−1^ for AOPMC at pH_eq_ = 5.3) for the experimental pH_eq_ range (1.0–5.3). In the studied pH range, both carbonaceous materials had a positively charged surface (pH < pH_PZC_). In the case of the OPMC material, practically all acidic oxygen-containing functionalities were protonated in these conditions. But for AOPMC material only -COOH groups were deprotonated, because the pK_a_ values for other acidic oxygen-containing groups and for amine groups originated from the L-arginine were greater than 5.3. Additionally, under experimental conditions, Pb(II) was present as Pb^2+^_(aq)_. Thus, repulsive electrostatic forces could occur between the carbonaceous surface and the studied ions during the adsorption process. This effect was the smallest at optimal pH_eq_ values. Not only electrostatic forces could influence the adsorption process of Pb(II) ions in the studied adsorption systems. Also, surface complexation (in both cases) and surface precipitation (for AOPMC) could be part of the adsorption mechanism [30,31,32].

**Figure 7:**
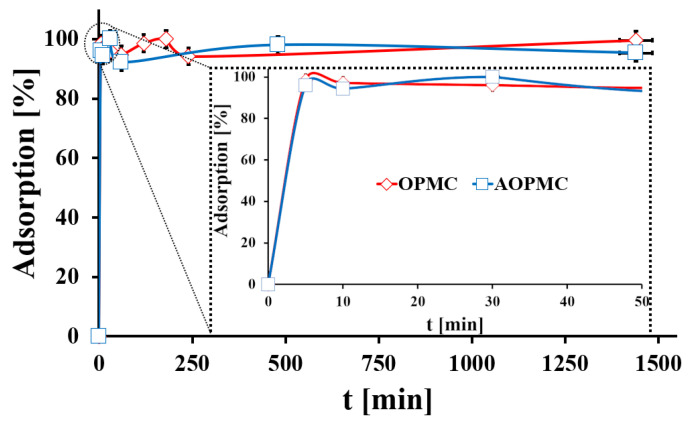
The Pb(II) adsorption kinetics onto studied materials (m/V = 4 g L^−1^, C_in_.__Pb(II)_ = 48 mg L^−1^, pH_eq_OPMC_ = 2.6., pH_eq_AOPMC_ = 5.3, A_100%_OPMC_ = 10.4 mg g^−1^, A_100%_AOPMC_ = 11.8 mg g^−1^; error bars—precision from triplicate measurements).

In Figure 7, the Pb(II) adsorption kinetics of both studied carbonaceous materials are presented. For both OPMC and AOPMC carbonaceous materials, the Pb(II) adsorption equilibrium state was achieved after 5 min. This fast adsorption equilibration was probably related to the high mass transfer through the ordered mesoporous channels of the studied materials. The Pb(II) adsorption kinetics data obtained were analyzed using three theoretical models: PFO, PSO, and Elovich [17,19] (Table 2). The PFO model best described the experimental data for OPMC and AOPMC (R^2^_OPMC_ = 0.957; R^2^_AOPMC_ = 0.973). In both cases, the Pb(II) diffusion to the surface active centers was the driving force for Pb(II) adsorption kinetics in the studied systems [33]. The similar k_1_ values for both materials suggested that the chemical surface modification did not influence the Pb(II) transfer to the adsorbent surface.

**Figure 8:**
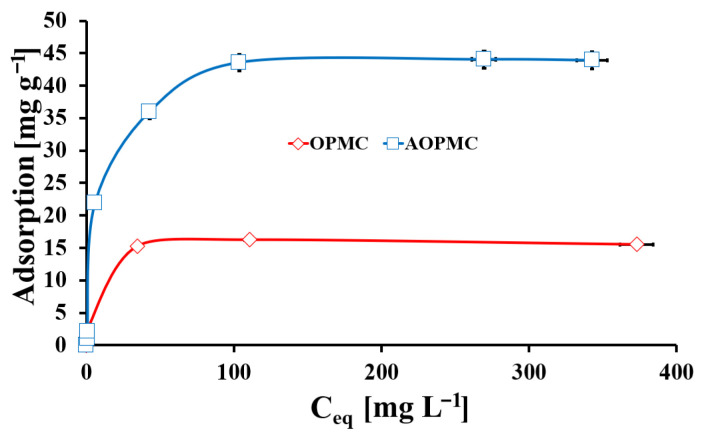
The Pb(II) adsorption equilibrium isotherms onto OPMC and AOPMC (m/V = 4 g L^−1^, pH_eq_OPMC_ = 2.6., pH_eq_AOPMC_ = 5.3, t_eq_ = 24 h, T = (25 ± 4) ^o^C; error bars—precision from triplicate measurements).

The adsorption equilibrium isotherms of Pb(II) ions for OPMC and AOPMC materials are depicted in Figure 8. The maximum adsorption capacities for Pb(II) ions of OPMC and AOPMC were 16 mg g^−1^ and 45 mg g^−1^, respectively. The modification of the OPMC surface by L-arginine resulted in a tripling of the maximum Pb(II) uptake capacity, probably due to an increase in the number of surface active sites. Moreover, the initial part of the Pb(II) adsorption isotherm for AOPMC material was located axially, which meant that for low Pb(II) concentrations, the high Pb(II) adsorption values were obtained. It could provide a higher enrichment factor for AOPMC material than for OPMC. So, the AOPMC material could be successfully applied to the analytical determination of Pb(II) ions. The obtained Pb(II) adsorption equilibrium data were analyzed using the following theoretical models: Langmuir, Freundlich, and Langmuir–Freundlich [17,20] (Table 3). The Langmuir model provided the best fit for both studied materials (R^2^_OPMC_ = 0.997, R^2^_AOPMC_ = 0.993). Additional confirmation of the best fitting of the Langmuir model was found in the g_m_ values, similar to the experimental maximum Pb(II) adsorption capacities (q_m_OPMC_ = 15.98 mg g^−1^, q_m_AOPMC_ = 44.94 mg g^−1^). It suggested chemisorption as the driving force for Pb(II) adsorption on both studied materials from aqueous solutions. The k_L_ values represented the Pb(II) affinity to the surface binding sites [34]. For OPMC material, the k_L_ value was higher (0.72 L mg^−1^) than for AOPMC material (0.14 L mg^−1^). It could be related to the stronger chemical forces between OPMC active sites and Pb(II) ions than between these ions and the AOPMC surface. Thus, the desorption of Pb from the AOPMC surface should be easier than in the case of Pb-loaded OPMC. Additionally, n_LF_ values for both materials were close to 1, indicating surface energetic homogeneity for both studied materials [20]. The Pb(II) adsorption performances of both synthesized carbonaceous materials were compared with other adsorbents in Table S2. For further studies, the AOPMC material was chosen due to its high Pb(II) adsorption capacity and the likely ease of regeneration.

### 3.3. Effect of Competing Ions

**Figure 9:**
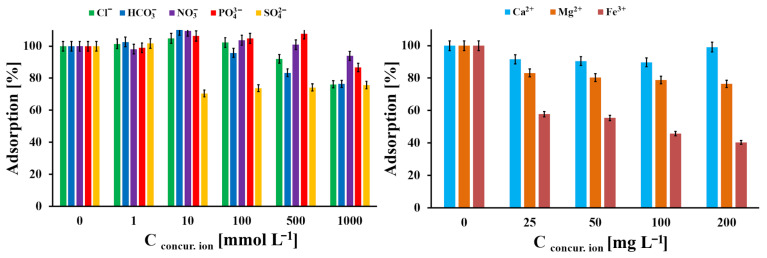
The influence of concurrent ions in studied adsorption system (m/V = 4 g L^−1^, C_in_.__Pb(II)_ = 36 mg L^−1^, pH_eq_ = 5.3, t_eq_ = 24 h, A_100%_ = 10.4 mg g^−1^; error bars—precision from triplicate measurements).

In Figure 9, the influence of concurrent ions in the adsorption system, Pb(II) and AOPMC, was studied. The noticeable interference was observed for SO_4_^2−^ at concentrations from 10 mmol L^−1^ (30% decrease in Pb(II) adsorption), for HCO_3_^−^ at concentrations from 500 mmol L^−1^ (20% decrease in Pb(II) adsorption), for PO_4_^3−^ and Cl^−^ at concentrations 1000 mmol L^−1^ (20% and 10% decrease in Pb(II) adsorption, respectively) and for Fe(III) at concentrations from 25 mg L^−1^ (40–60% decrease in Pb(II) adsorption). The Fe(III) ions, which interfered the most in the studied adsorption system, could be complexed to a greater extent than Pb(II) ions by the AOPMC surface, due to its higher electrostatic charge and greater affinity towards amine and carboxyl ligands. In turn, the SO_4_^2−^ ions could form the hardly soluble salt (PbSO_4_) with Pb(II) ions, and thus they could be concurrent for the adsorbed ions.

### 3.4. Mechanism of Adsorption

The discovery of the Pb(II) adsorption mechanism onto AOPMC carbonaceous material was performed by the XPS study of the Pb-loaded AOPMC. The elemental surface composition of the Pb-loaded AOPMC was presented in Table S3. The following could be indicated after comparing these data with XPS data for the pristine AOPMC material: during the Pb(II) adsorption onto the studied material, the C content declined (from 83.1 wt. % to 68.9 wt. %), the O content improved (from 9.9 wt. % to 15.3 wt. %), the N content did not change, and the Pb appeared (8.2 wt. %). These changes could indicate partial AOPMC surface oxidation by HNO_3_ in aqueous Pb(II) solution, surface complexation, or precipitation of Pb(II) as hydroxide. More information was obtained from studies of the high-resolution XPS core energy levels: C1s, O1s, N1s and Pb4f. The results of the mathematical deconvolution of the mentioned XPS signals are presented in Table S4. The partial oxidation of the AOPMC surface by the HNO_3_ was confirmed (the participation increase in C=O, O=C-O, O-C_aliph_., O-C_arom_., -NO_2_ and CO_3_^2−^ in the intensities of C1s, O1s and N1s XPS signals). Additionally, the appearance of the O^2−^, metal oxide peak in the O1s XPS signal and Pb(NO_3_)_2_ peak in the Pb4f XPS signal suggested the presence of Pb-O bonds on the AOPMC surface. Thus, the Pb(II) could be adsorbed onto the AOPMC surface during the surface complexation by the N and O surface functionalities and/or surface precipitation in the form of the Pb(OH)_2_.

### 3.5. Reusability Studies

**Figure 10:**
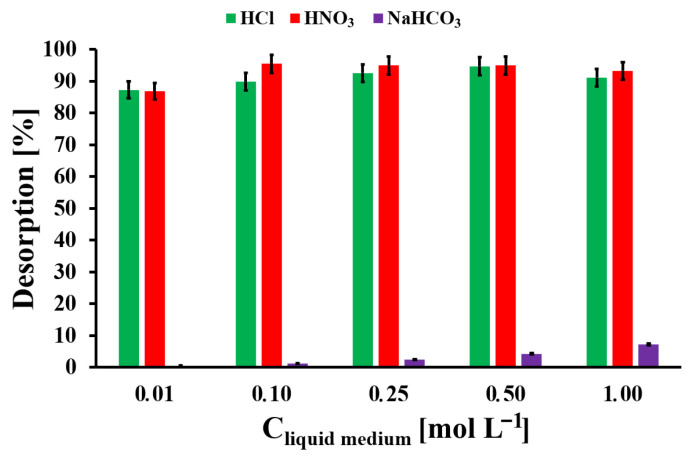
The Pb desorption studies from Pb-loaded AOPMC material (A_Pbcarb_. = 41.2 mg g^−1^, m_des_. = 5 mg, V_lm_ = 2 mL, t_des_. = 24 h; error bars—precision from triplicate measurements).

Also, studies on the reusability of the Pb-loaded AOPMC material were conducted using three different liquid media: HCl, HNO_3_, and NaHCO_3(aq)_ (Figure 10). The best liquid medium, which practically provided the quantitative (100%) Pb desorption from the Pb-loaded AOPMC, was 0.1 mol L^−1^ HNO_3_. Similar results were obtained for HCl. It could be the result of the AOPMC surface protonation by the acid and the electrostatic repulsion of the adsorbate ions from the positively charged carbonaceous surface. In turn, the NaHCO_3(aq)_ resulted in a low extent of Pb desorption (<5%). For further studies, 0.1 mol L^−1^ HNO_3_ was used, due to the absence of interference in the GFAAS technique (unlike HCl) and its sufficiently low concentration to avoid rapid corrosion of the graphite cuvette.

### 3.6. Pb(II) Determination in Simulated Urban Runoff by SS HR-CS GF AAS Technique

The AOPMC carbonaceous material was studied for Pb(II) enrichment from simulated urban runoff and its determination by SS HR-CS GF AAS technique. In order to verify whether the calibration graph based on aqueous Pb standard solutions could be used during determinations by the SS HR-CS GF AAS technique, the absorbance signals for the same Pb amount injected into the graphite tube from the aqueous solution and the AOPMC slurry were compared (Figure S1). The obtained absorbance signals were similar, suggesting that the proposed calibration strategy would be appropriate. The following analytical parameters of the proposed procedure were estimated: the calibration graph linear range of 4–40 µg L^−1^, the limit of quantification of 2.63 µg L^−1^ (based on 10 standard deviations of the absorbance for the blank—the AOPMC carbonaceous slurry without adsorbed Pb(II) in 0.1 mol L^−1^ HNO_3_–and the slope of the calibration graph) and the recovery of 94.8%. Additionally, the enrichment factor for the proposed analytical procedure was estimated as the ratio of the Pb mass introduced to the graphite atomizer along with the AOPMC carbon to the Pb mass introduced to the graphite atomizer directly from the analyzed sample solution. For the proposed procedure, the enrichment factor of 50 was obtained. In Table 4, the results of Pb determination in the simulated urban runoff samples and SRM1640, using the SS HR-CS GF AAS technique after Pb enrichment with the AOPMC material, are presented. It could be seen that the AOPMC material was successfully applied to the proposed analytical application.

## 4. Conclusions

The novel highly porous (>400 m^2^ g^−1^) hexagonally ordered mesoporous carbon modified with L-arginine was successfully synthesized and characterized by various physicochemical methods. The L-arginine molecules and their polymeric derivatives were partially incorporated inside the mesoporous channels of the material. The nearly neutral surface of the synthesized material contained both oxygen and nitrogen heteroatoms, which could bond with heavy metal ions such as Pb(II).

The highest adsorption capacity towards Pb(II) ions for the studied material (45 mg g^−1^) was achieved at pH = 5.3 only after 5 min. The adsorption kinetics were best described by the pseudo-first-order model, suggesting that the diffusion of Pb(II) toward the active surface sites of the studied material controlled the adsorption process. The adsorption isotherm was best fitted by the Langmuir model, indicating that Pb(II) adsorption on the surface of the L-arginine-modified carbonaceous material proceeds via chemisorption.

The adsorption of Pb(II) ions using the synthesized material was driven by surface complexation via nitrogen- and oxygen-containing surface functionalities and/or by surface precipitation as Pb(OH)_2_.

The material with adsorbed Pb could be easily regenerated with 0.1 mol L^−1^ HNO_3_, which protonated the carbonaceous surface and enabled electrostatic repulsion of the adsorbed Pb(II) ions from the material surface. It could substantially decrease the costs of routine analyses by using this material during the enrichment step.

The L-arginine-modified ordered mesoporous carbon was successfully applied to Pb(II) determination in simulated urban runoff using the SS HR-CS GF AAS technique (recovery of 94.8%). It could be used for HRW routine quality control of Pb(II) concentration.

## Figures and Tables

**Table 1:** Porosity characteristics (S_BET_, d_BJH_, V_Tot_.), Raman factor (I_D_/I_G_), pH_PZC_ and ζ-potential for OPMC and AOPMC carbonaceous materials.

Material Symbol	S_BET_ [m^2^ g^−1^]	d_BJH_ [nm]	V_Tot_. [cm^3^ g^−1^]	I_D_/I_G_ [a. u.]	pH_PZC_ [a. u.]	ζ [mV]
OPMC	813 * ± 15 ^&^	3.4 * ± 0.1 ^&^	0.82 * ± 0.01 ^&^	0.86 * ± 0.01 ^&^	2.9 * ± 0.03 ^&^	−33.7 * ± 0.1 ^&^
AOPMC	430 * ± 10 ^&^	3.4 * ± 0.1 ^&^	0.43 * ± 0.01 ^&^	0.87 * ± 0.03 ^&^	6.53 * ± 0.14 ^&^	−33.1 * ± 0.1 ^&^

*—the mean from triplicate measurements; ^&^—the precision from triplicate measurements

**Table 2:** Results of the non-linear fitting of the Pb(II) adsorption kinetics data to PFO, PSO and Elovich models for OPMC and AOPMC.

Material Symbol	PFO			PSO			Elovich		
	g_eq_t_ [mg g^−1^]	k_1_ [min^−1^]	R^2^	g_eq_t_ [mg g^−1^]	k_2_ [g mg^−1^ min^−1^]	R^2^	α [mg g^−1^ min^−1^]	β [g mg^−1^]	R^2^
OPMC	10.50 * ± 0.68 ^#^	0.42 * ± 0.09 ^#^	0.957	11.83 * ± 0.48 ^#^	0.045 * ± 0.001 ^#^	0.901	15.59 * ± 1.40^#^	0.45 * ± 0.06^#^	0.813
AOPMC	11.96 * ± 0.62 ^#^	0.40 * ± 0.07 ^#^	0.973	13.55 * ± 0.38 ^#^	0.037 * ± 0.003 ^#^	0.933	16.90 * ± 3.60 ^#^	0.39 * ± 0.01 ^#^	0.862

*—the mean from triplicate measurements; ^#^—the precision from triplicate measurements.

**Table 3:** Results of the non-linear fitting of the Pb(II) adsorption equilibrium isotherms to Langmuir, Freundlich and Langmuir–Freundlich models for OPMC and AOPMC.

Material Symbol	Langmuir			Freundlich			Langmuir–Freundlich			
	g_m_ [mg g^−1^]	k_L_ [L mg^−1^]	R^2^	n_F_ [a. u.]	k_F_ [mg^(1−nF)^ L^nF^]	R^2^	n_LF_ [a. u.]	g_m_ [mg g^−1^]	k_LF_ [L mg^−1^]	R^2^
OPMC	15.98 * ± 0.26^#^	0.72 * ± 0.14 ^#^	0.997	0.19 * ± 0.01 ^#^	5.82 * ± 0.90 ^#^	0.866	0.95 * ± 0.09 ^#^	16.07 * ± 0.48 ^#^	0.65 * ± 0.08 ^#^	0.996
AOPMC	44.94 * ± 1.09^#^	0.14 * ± 0.02 ^#^	0.993	0.25 * ± 0.02 ^#^	11.22 * ± 0.48 ^#^	0.886	1.00 * ± 0.16 ^#^	44.51 * ± 1.70 ^#^	0.14 * ± 0.03 ^#^	0.991

*—the mean from triplicate measurements; ^#^—the precision from triplicate measurements.

**Table 4:** Results of the Pb(II) determination in simulated urban runoff and SRM1640 by SS HR-CS GF AAS.

Sample Symbol	Z_Pb_ [µg L^−1^]	Reference Concentration [µg L^−1^]
S1	4.23 * ± 0.30 ^#^	-
S2	7.87 * ± 0.50 ^#^	-
S3	11.91 * ± 0.51 ^#^	-
SRM1640	26.43 * ± 0.47 ^#^	27.89 * ± 0.14 ^#^

*—the mean from triplicate measurements; ^#^—the precision from triplicate measurements.

## Data Availability

The original contributions presented in the study are included in the article/Supplementary Materials. Further inquiries can be directed to the corresponding author.

## Supplementary Materials

The following supporting information can be downloaded at: https://www.mdpi.com/article/10.3390/ma19071318/s1, Table S1: The composition of the matrix reference material Rain-97; Table S2: The comparison of Pb(II) adsorption performances for mesoporous carbon materials; Table S3: The elemental XPS composition of the Pb-loaded AOPMC material (Pb content: 50 mg/g); Table S4: The participation of the deconvoluted XPS peaks in the intensity of C1s, O1s, N1s and Pb4f XPS signals for Pb-loaded AOPMC material (Pb content: 50 mg g^−1^); Figure S1: The GF AAS signals for Pb standard solution (10 µg/L) and AOPMC slurry containing Pb (2.14 µg/g).
