# Supplementary material for: Ordered Mesoporous Carbon Modified with L-Arginine for Pb(II) Enrichment in Water Quality Control from Urban Roof Runoff
Source: Materials (Basel). 2026 Mar 26;19(7):1318. doi: 10.3390/ma19071318 (PMC13074665; doi:10.3390/ma19071318)
Supplement: Supplementary file 1 [file materials-19-01318-s001.zip › materials-4197336-supplementary.pdf]

SUPPLEMENTARY

Ordered mesoporous carbon modified with L-arginine as efficient Pb(II) adsorbent for water quality control from urban roof runoff

Rafał Olchowski [1], Agnieszka Chałabis-Mazurek [1] and Ryszard Dobrowolski [2,*]

[1] Department of Pharmacology, Toxicology and Environmental Protection, Faculty of Veterinary Medicine, University of Life Sciences, Akademicka Sq. 12, Lublin, 20-950, Poland; rafal.olchowski@up.lublin.pl
[2] Department of Analytical Chemistry, Faculty of Chemistry, Institute of Chemical Sciences, Maria Curie-Sklodowska University, M. C. Sklodowska Sq. 3, Lublin, 20-031, Poland;
* Correspondence: ryszard.dobrowolski@mail.umcs.pl

Table S1. The composition of the matrix reference material Rain-97 [21].

**Component**	**Concentration ± SD [mg L^{-1}]**
Na^+	0.298 ± 0.029
NH_4^+	0.186 ± 0.024
K^+	0.166 ± 0.026
Ca^{2+}	2.860 ± 0.203
Mg^{2+}	1.000 ± 0.078
Cl^-	0.574 ± 0.058
$NO_3^- + NO_2^-$	2.220 ± 0.120
SO_4^{2-}	5.680 ± 0.350

Table S2. The comparison of Pb(II) adsorption performances for mesoporous carbon materials.

**Material description**	**Pb(II) adsorption performance**	**Literature**
OPMC (ozonized CMK-3)	$pH_{opt.}$ = 2.6, t_{eq} = 5 min, a_{max} = 16 mg g^{-1}	present work
AOPMC (L-arginine modified CMK-3)	$pH_{opt.}$ = 5.3, t_{eq} = 5 min, a_{max} = 45 mg g^{-1}	
MOMC-NP (ordered mesoporous carbon modified by nitric and orthophosphoric acids)	$pH_{opt.}$ = 5.0, t_{eq} = 25 min, a_{max} = 120.6 mg g^{-1}	[1]
BMC (boron-doped CMK-3)	t_{eq} = 60 min, a_{max} = 415.5 mg g^{-1}	[2]
COMC (ordered mesoporous carbon oxidized by nitric acid)	$pH_{opt.}$ = 7.0, t_{eq} = 360 min, a_{max} = 333.3 mg g^{-1}	[3]

[1] https://doi.org/10.1007/s11356-020-08487-9
[2] https://doi.org/10.1016/j.scitotenv.2019.134918
[3] https://doi.org/10.1016/j.apsusc.2009.08.106

Table S3. The elemental XPS composition of the Pb-loaded AOPMC material (Pb content: 50 mg/g).

Element	Concentration ± SD [wt. %]
C	68.9 ± 1.4
O	15.3 ± 0.2
N	6.7 ± 0.2
Pb	8.2 ± 0.3

Table S4. The participation of the deconvoluted XPS peaks in the intensity of C1s, O1s, N1s and Pb4f XPS signals for Pb-loaded AOPMC material (Pb content: 50 mg g^{-1}).

XPS deconvoluted peak (binding energy [eV])	Participation [%]
**C1s**	
C=Csp^2 (284.6)	43.7
C-Csp^3 (285.4)	31.5
C-O/C-N (286.5)	9.3
C=O (287.8)	6.6
O=C-O (289.0)	6.4
CO_3^{2-} (290.7)	2.5
**O1s**	
O^{2-}, metal oxide (530.6)	4.7
<u>O</u>=C-O (531.5)	32.4
O-$C_{aliph.}$ (532.6)	35.2
O-$C_{arom.}$ (533.7)	23.6
$H_2O/O_{2\ ads.}$ (535.6)	4.2
**N1s**	
=N- (399.3)	20.8
-NH- (400.4)	65.0
$-NO_2$ (406.8)	14.2
**Pb4f**	
$Pb(NO_3)_2$ (139.0, 143.9)	100

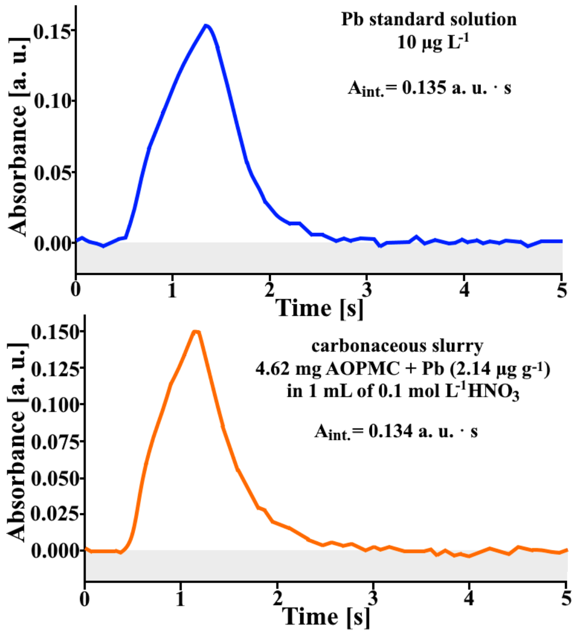

Figure S1. The GF AAS signals for Pb standard solution (10 μg L^{-1}) and AOPMC slurry containing Pb (2.14 μg g^{-1}).
